# Systematic review on antigens for serodiagnosis of visceral leishmaniasis, with a focus on East Africa

**DOI:** 10.1371/journal.pntd.0007658

**Published:** 2019-08-15

**Authors:** Vera Kühne, Zahra Rezaei, Paul Pitzinger, Philippe Büscher

**Affiliations:** 1 Department of Biomedical Sciences, Institute of Tropical Medicine, Antwerp, Belgium; 2 Professor Alborzi Clinical Microbiology Research Center, Shiraz University of Medical Sciences, Shiraz, Iran; 3 Institute of Medical Microbiology, University Medical Center Göttingen, Göttingen, Germany; RTI International, UNITED STATES

## Abstract

**Background:**

Accurate and accessible diagnosis is key for the control of visceral leishmaniasis (VL). Yet, current diagnostic tests for VL have severe limitations: they are invasive or not suitable as point of care (POC) test or their performance is suboptimal in East Africa. We analysed the antigens in the VL serodiagnostics development pipeline to identify shortcomings and to propose strategies in the development of an alternative POC test for VL in East Africa.

**Objectives:**

The objective of this study was to identify and to analyse all antigens for VL serodiagnosis that have been published before 2018 in order to identify candidates and gaps in the pipeline for a new POC test in East Africa.

**Methods:**

A systematic literature search was performed on PubMed for original research articles on *Leishmania*-specific antigens for antibody detection of VL in humans. From each article, the following information was extracted: the antigen name, test format and characteristics, its reported sensitivity and specificity and study cohort specifications.

**Results:**

One hundred and seven articles containing information about 96 tests based on 89 different antigens were included in this study. Eighty six of these tests, comprising 80 antigens, were evaluated in phase I and II studies only. Only 20 antigens, all of which are native, contain a carbohydrate and/or lipid moiety. Twenty-four antigens, of which 7 are non-native, are composed of antigen mixtures. Nineteen tests, comprising 18 antigens, have been evaluated on East African specimens, of which only 2 (rK28 based immunochromatographic test and intact promastigote based indirect fluorescent antibody technique) consistently showed sensitivities above 94 and specificities above 97% in a phase III study and one in a phase II study (dot blot with SLA). Only rK28 is a non-native mixture of antigens which we consider suitable for further evaluation and implementation.

**Conclusions:**

The development pipeline for an alternative serodiagnostic test for VL is almost empty. Most antigens are not sufficiently evaluated. Non-protein antigens and antigen mixtures are being neglected. We propose to expand the evaluation of existing antigen candidates and to investigate the diagnostic potential of defined non-native carbohydrate and lipid antigens for VL serodiagnosis in East Africa.

## Author summary

Visceral leishmaniasis is a potentially fatal disease that affects more than 20 000 people every year. Its diagnosis is difficult since the clinical symptoms are not specific and the existing diagnostic tests are not useful in limited resource countries or they a not accurate enough in East Africa. In this review we performed a systematic search of the published literature to analyse the potential candidate antigens in the pipeline for a new antibody detection test in East Africa. We found 96 tests based on 89 antigens. Eighty six of these tests were evaluated in a study design that is insufficient (phase I and II) to make conclusions on their performance in clinical practice. We found that the candidate antigens either lacked carbohydrate or lipid structures or are based on single antigens as opposed to mixtures or are extracted from the causative parasite itself, making them expensive and prone to variations. Considering that the most widely used diagnostic test does not detect all cases of visceral leishmaniasis in East Africa, we analysed how many of the candidate antigens were tested on East African specimens: We found that only 2 tests (rK28 based immunochromatographic test and the intact promastigote based indirect fluorescent antibody technique) that were tested in a phase III study and only one (dot blot with SLA) that was tested in a phase II study performed well according to our criteria. Due to the antigen characteristics we consider only the rK28 based test as suitable for further evaluation and implementation.

## Introduction

### Rationale

Leishmaniasis comprises a group of diseases caused by protozoan parasites of the genus *Leishmania* that are transmitted between human or other mammalian hosts by phlebotomine sand flies [1]. Visceral leishmaniasis (VL) is the most severe form of the disease, for it is almost always fatal if left untreated. In 2016, VL was reported in 21646 cases worldwide, while the estimated number of underreported cases is high (up to 85% in 2012) [2,3]. The typical causative agent of VL is the *Leishmania (L*.*) donovani* complex, which includes the two species *L*. *donovani* and *L*. *infantum*. On the Indian subcontinent and in East Africa, *L*. *donovani* is almost exclusively the cause of VL[4,5]. Anti-leishmaniasis drugs are toxic, expensive and/or prone to induce drug resistance.

The gold standard for diagnosis of VL remains the microscopic detection of the parasite in patient tissues. The most sensitive technique requires a splenic puncture, while lymph node and bone marrow aspirates can also be used with lower sensitivity [5,6]. These invasive diagnostics have several disadvantages, as they require highly skilled medical personnel as well as suitable clinical facilities and equipment. Furthermore, splenic and bone marrow aspirates are painful and possibly dangerous.

Less invasive techniques applicable on blood exist. Due to their labour-intensiveness and their technical requirements, molecular diagnostic tests as well as other laboratory tests, such as ELISA, are not applicable in the field [5]. The ideal diagnostic test for limited resource settings should comply with the ASSURED criteria: be accurate, sensitive, specific, user friendly, rapid and robust, equipment-free, and delivered to those who need it[7]. In this regard the immunochromatographic rapid diagnostic test (RDT) with rK39 antigen was a major breakthrough in terms of access to VL diagnosis for populations at risk [8]. It has become the reference test for VL on the Indian subcontinent. It even has potential use to monitor treatment outcome when used to detect IgG1 [9]. The rK39 consists of 6,4 repeats of a 39-amino acids stretch belonging to a kinesin-related protein of *L*. *chagasi* expressed in *Escherichia (E*.*) coli* [8].

Unfortunately, the diagnostic accuracy of rK39-based RDTs has been shown to be dependent on the geographic region. While high sensitivities have been reported in Indian populations (97.0%; 95% CI 90.0 to 99.5), reported sensitivities in East African populations are variable and generally lower (85.3%; 95% CI 74.5 to 93.2) [5,6,10–12].

In 2010, an RDT with rK28 as antigen has been developed to overcome the issue of low sensitivity of rK39-based tests in Eastern Africa. rK28 is a chimeric antigen composed of three 14-amino acid repeats of the *L*. *donovani* HASPB1 gene, two 39 amino acid repeats of the *L*. *donovani* K39 kinesin protein gene and the complete open reading frame of *L*. *donovani* hydrophilic acylated surface protein B2 (HASPB2) gene expressed in *E*. *coli* [13]. RDTs with rK28 antigen have been evaluated in four studies in Sudan and Ethiopia with promising results for the test manufactured by CTK Biotech (sensitivities between 94 and 98%, and specificities between 95 and 98%) while sensitivities and specificities based on RDTs by other manufacturers and in ELISA were more variable (sensitivities between 89 and 100%, and specificities between 81 and 99%) [13–17].

With rk28 having been evaluated on a relatively small number of specimens (VL suspected patients: 285), there is a risk that its performance turns out to be variable based on the population it is evaluated on. For this reason we find it important that alternative antigens are investigated. We analysed the antigens in the pipeline, to identify candidate antigens, potential gaps in the development process and to propose alternative strategies towards improved serodiagnostics for VL in East Africa.

### Objectives

Our objective was to analyse all the published studies on serodiagnostic antigens for VL, regarding their characteristics, the test format, the study design, the number and geographic origin of tested specimens and the reported diagnostic accuracy.

## Materials and methods

A systematic literature search was performed on PubMed on 21 March 2018 of all articles published until 2018, using the string “((((diagnostic) AND antigen) AND visceral) AND leishmaniasis) NOT vaccine”. As we noticed that this search string was missing articles describing antigens which were analysed as both a vaccine candidate and diagnostic antigen a next screen was performed with the search string "((((diagnostic) AND antigen) AND visceral) AND leishmaniasis) AND vaccine". Results of both search strings were merged and analysed together. Titles and abstracts were screened by two independent investigators (VK&ZR). In case of non-concordance, a third independent investigator (PP) screened title and abstract of the respective articles. Both the second and third investigator were blinded to the other investigators’ decisions. If the first investigator contested the second and third investigators decision, a senior investigator (PB) was consulted for a final decision. There was no formal study protocol and the study was not registered.

Based on information in the title and abstract, all original research articles that reported on the diagnostic potential of one or more molecules for antibody detection of VL in humans, were included. Systematic reviews and meta-analyses on such articles were also included. Exclusion criteria were: i) only non-human (e.g. canine) specimens (serum or plasma) were tested, ii) only non-VL specimens (e.g. tegumentary, cutaneous, mucocutaneous leishmaniasis) were tested, iii) study on non-antibody detection tests (e.g. molecular, microscopic, antigen detection), iv) study on antigens that were not used for serodiagnosis of VL (e.g. test for experimental infection or treatment outcome or for epidemiological surveys), v) study on immunological biomarkers such as cytokines. The remaining articles were analysed based on their full text. Additional exclusion criteria were: i) no control specimens were tested (other disease or healthy endemic controls); ii) less than 5 specimens tested; iii) antigen with poor diagnostic potential (< 50% sensitivity and/or < 50% specificity); iv) data for sensitivity and specificity not presented; v) number or geographic origin of specimens not described vi) antigen not *Leishmania-*specific (e.g. BCG, Kendrows Bacillus); vii) article written in a language other than English, French or German; viii) full article text not available. Antigens (rK39 and stained formaldehyde-fixed promastigotes for direct agglutination test [DAT]) that were already included in a meta-analysis by Chappuis et al. (2006) [10] or Boelaert et al. (2014) [6] were not re-analysed but the results of the meta-analysis were taken into account [6,10].

For each included article, the following data were imported in a Microsoft Excel worksheet: antigen name, molecular weight, reported sensitivity and specificity, study design, number or VL cases and controls tested, type of assay, type of controls tested, type of specimen tested, geographical origin of the tested specimens, *Leishmania* species and strain from which the antigen was derived, method of antigen preparation/production, article reference. The individual studies were not analysed for risk of bias as we intended to analyse the complete data on antibody detectiontests for VL including early discovery studies with limited number of specimens and high risk of bias. We did not perform a meta-analysis on the diagnostic sensitivity and specificity of the tests, which reduces the potential bias of the individual studies.

The data were analysed in three ways:

1. Based on the study design and the number of specimens used for evaluation, the studies were classified according to the three phases in diagnostic evaluation test for VL as defined by Boelaert and co-workers in 2007 [18,19]. The following phases were defined: phase I, exploratory study to provide proof-of-principle on 10–100 specimens; phase II, case-control study on >100 specimens; phase III, large-scale prospective study on target population, usually on a minimum of 300 persons. Studies within the same phase were grouped by test and specimens used for evaluation were cumulated per test. For phase III studies, tests were then divided by the cumulative number of persons tested: < 600; 601 - ≤ 5000 and > 5000. Sensitivities and specificities of the same test in different studies in the same phase were merged and the lowest and highest values were used for the range. For the meta-analysis reports, the confidence intervals are considered as the range. Tests were analysed on whether the range of sensitivities and specificities per phase met the cut off of >94% sensitivity and >97% specificity for an ideal test defined by Boelaert et al, 2007 [18].
2. The characteristics of the antigens. Based on the preparation and/or the known chemical composition of the antigens, they, were classified as follows: a) certainly contains no carbohydrate or lipid moiety (recombinantly expressed in *E*. *coli* or phages, synthetic peptides or antigens analysed for non-protein moieties), b) contains a carbohydrate moiety, c) contains a carbohydrate and lipid moiety, d) unknown whether it contains a carbohydrate or lipid moiety. Moreover, antigens were analysed based on whether they could be attributed one or more of the following characteristics: a) contains a carbohydrate and/or lipid moiety, b) composed of a mixture of antigens (including chimeric recombinant antigens), c) native antigen, d) antigen defined by its annotation or by its isolation process and subsequent characterisation.
3. Availability of data on diagnostic evaluation with East African specimens.

## Results

**Fig 1:**
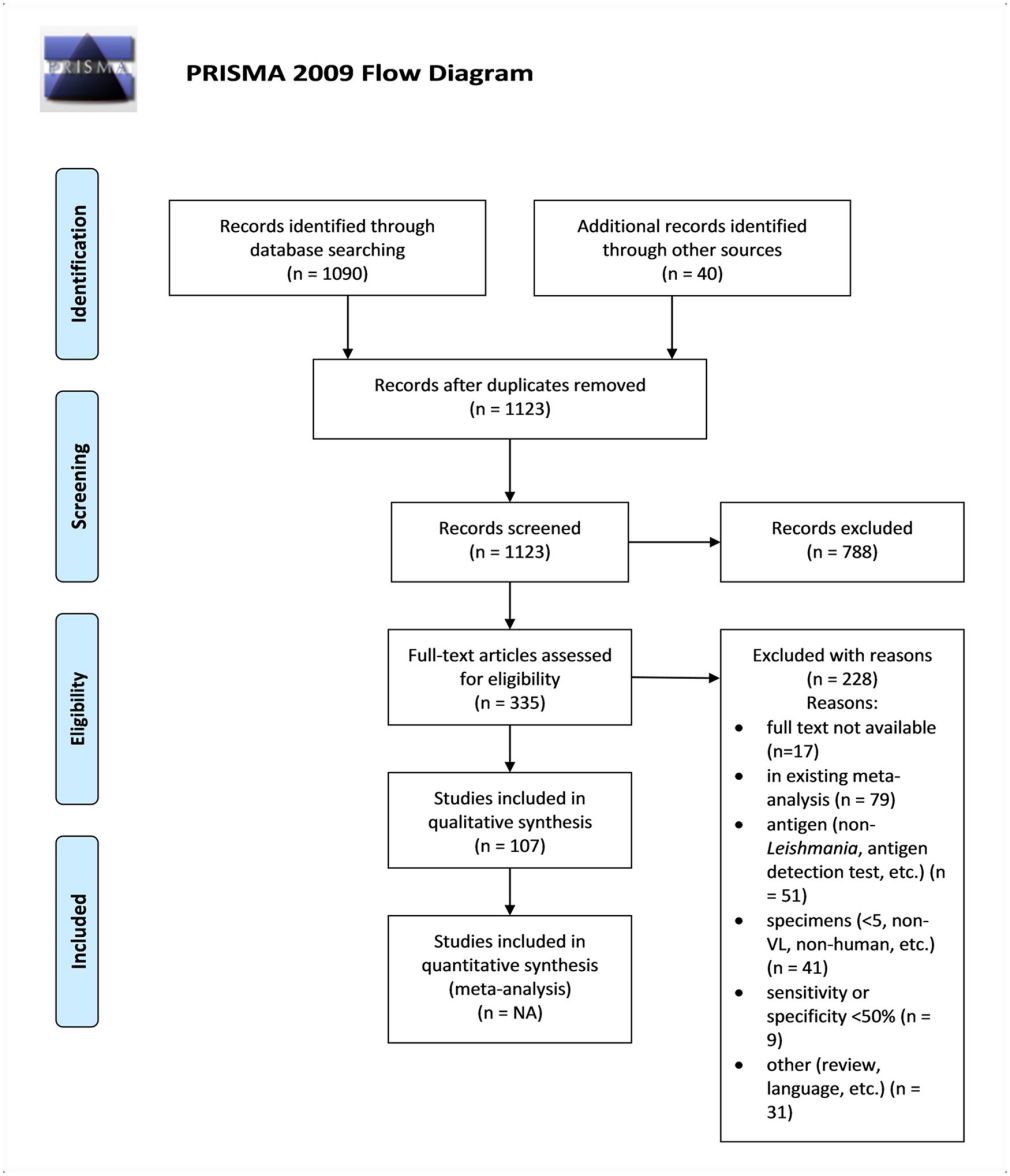
PRISMA 2009 flow diagram adapted from Moher et al. (2009) [20].

The search resulted in 1123 non-duplicate articles, of which 107 were eventually eligible according to abstract screening and full-text review (Fig 1 for PRISMA diagram). The two hundred and thirteen discordant decisions regarding the inclusion of articles between first and second investigator were screened by the third investigator. In all 28 cases in which the first investigator contested the third investigator’s decision, this was confirmed by the senior investigator. Of 335 articles, 228 were excluded based on the full text screening. The main reasons for exclusion in the full text screen were the following: full text not available (n = 17), the article was already included in an existing meta-analysis by Chappuis 2006 [10] or Boelaert 2014 [6] (n = 79), the antigen did not comply with the eligibility criteria (e.g. non-*Leishmania*, for antigen detection) (n = 51), specimens used for evaluation did not conform to the eligibility criteria (e.g. <5, non-VL or non-human specimen tested) (n = 41) or the diagnostic sensitivity and/or specificity was <50% (n = 9). The extracted data on antigen name, molecular weight, reported sensitivity and specificity, number or VL cases and controls tested, type of assay, type of controls tested, type of specimen tested, geographical origin of the tested specimens, *Leishmania* species and article reference can be found in the supporting information (S1 Table).

### The position of the antigens along the diagnostic development pipeline

**Fig 2:**
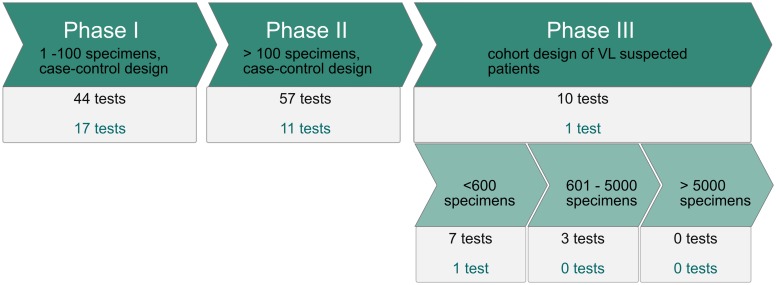
Serodiagnostic tests for VL in the different phases of the diagnostic development pipeline. Each test is defined by its antigen and the test format. The number of tests shown in black is the total number of tests that have been evaluated in this phase. The number of tests in black gives the total number of tests per phase, the number of tests shown shown in green gives the number of tests that meet the cut off of >94% sensitivity and >97% specificity for an ideal test defined by Boelaert *et al*, 2007 [18].

Data from the included articles were grouped per type of test, test antigen and study design (phase I, II and III). For studies on the same test in the same phase, the total numbers of cases and controls were calculated. We found 96 serodiagnostic tests comprising 89 antigens for human VL (Fig 2, Table 1). As Bhattacharyya *et al*. pointed out in their recent review, the use of "rk" in the antigen nomenclature is misleading [21]. Eight different antigens included in our analysis are based on genes for kinesin related proteins such as rk39 (Table 2).

**Table 1:** Detailed overview of serodiagnostic tests and antigens for VL in the diagnostic development pipeline. For tests evaluated in multiple studies the lowest and highest values are taken for the sensitivity and specificity range. For the meta-analysis reports, the confidence intervals are considered as the range. Highlighted in grey are the tests that meet the cut off of >94% sensitivity and >97% specificity for an ideal test defined by Boelaert et al, 2007 [25]. ß -ME: beta-mercaptoethanol, DAT: Direct Agglutination Test, ELISA: enzyme-linked immunosorbent assay, FACS: fluorescence-activated cell sorting, ICT: immunochromatographic test, IFAT: indirect fluorescent antibody technique, kDa: kilodalton, r: recombinant, SDS-PAGE: sodium dodecyl sulfate polyacrylamide gel electrophoresis, SLA: soluble leishmanial antigen, WB: Western blot.

	**phase I**						**phase II**					
**Test format**	**antigen name**	**number of studies**	**number of specimens tested**	**reported sensitivity [%]**	**reported specificity [%]**	**ref**.	**antigen name**	**number of studies**	**number of specimens tested**	**reported sensitivity [%]**	**reported specificity [%]**	**ref**.
**ICT**	OrangeLife (an rK39 + rK28 ICT)	1	95	80	98	[22]	LAg	2	454	100	100	[23,24]
	P1P2	1	50	100	95	[26]	rK28	3	629	94–99 (CTK: 94–98)	81–95 (CTK: 95–99)	[13,14]
	rKE16	1	95	100	97	[27]	rKE16	2	1349	37–96	95–100	[12,28]
**ELISA**	62 to 63 kDa Protein	1	30	100	100	[30]	A2-MBP	1	146	57	90	[31]
	66 kDa antileishmanial antigen	1	72	97	97	[34]	acetone-treated urine ELISA	1	276	95	95	[35]
	70 kDa soluble antigen	1	42	100	100	[38]	amastigote SLA	1	206	94	92	[39]
	70-P2	1	33	57	100	[40]	BHUP1	1	277	95	100	[41]
	B10 peptide	1	92	91	90	[42]	BHUP2	1	172	94	97–100	[43]
	B10 phage	1	92	100	98	[42]	BHUP3	1	413	88	96	[44]
	C01 peptide	1	92	92	86	[42]	B-ME ELISA	1	314	93	96	[45]
	C01 phage	1	92	92	98	[42]	c-ELISA D2	1	181	100	96	[46]
	C9 antigen	1	90	68	78	[47]	CLH	1	177	98	100	[48]
	c-ELISA D2+D13+D14	1	77	90	100	[49,50]	ESAs	3	380	100	95–100	[51–53]
	CPB and CPA	1	25	11 rCPA, 76 rCPB	88 rCPA, 50 rCPB	[57]	gp70-2	1	126	90	98	[55,56]
	dp 72	1	92	100	93	[55,56]	Intact promastigote ELISA	1	135	100	not calculated	[58]
	ESAs	1	26	100	100	[52]	K9-K26-K39	1	365	82	99	[59]
	Intact promastigote ELISA	2	161	69–100	88–100	[61,62]	LAg	1	85	100	97	[60]
	LBPs	1	53	100	93	[63]	LPG	1	146	92	92	[31]
	LicTXNPx	1	45	78	94	[65]	MAPK3	1	125	95	31	[64]
	Lmjsp	1	15	100	100	[66]	MAPK4	1	125	73	89	[64]
	mimotopes	1	57	100	100	[67]	P20	1	146	68	95	[31]
	peptides A2, NH, LACK and K39	1	44	100	100	[69]	P32	1	152	94	94	[68]
	peptides gp63	1	100	all 5: 97, P2+P4/P3: 100	all 5: 76, P2+P4/P3: 94	[71]	p-ELISA	1	1134	87	93	[70]
	Q protein	1	81	100	100	[73]	r(g)p63	2	276	84–86	97–98	[31,72] [72]
	rA2	1	55	70	100	[76]	rA2	2	256	65–78	74–92	[74] [75]
	rH2A	1	81	100	100	[73]	rBHUP1	1	495	97	96, OD: 75	[77]
	rK26	1	80	38	80	[79]	rCatL	1	195	75	91	[78]
	rLdRab6	1	68	100	100	[80]	rCatL epitope Peptide 1	1	195	95	97	[78]
	rLiHsp70	1	33	79	14	[40]	rDD8	1	469	100	98	[81]
	rLmSODB1	1	65	63	98	[82]	rGBP	1	146	97	92	[31]
	rORFF	1	87	100	87	[83]	rH2A	1	146	100	91	[31]
	rSGT	1	97	100	100	[84]	rH2B	1	146	100	92	[31]
	SLA + intact promastigote	1	67	ELISA +FACS: 100	ELISA: 77 FACS: 83	[86]	rHSP83	1	139	100	93	[85]
	SLA/ Lysate	4	253	75–98	63–97	[42,62,88,89]	rHSP83.1	1	125	53	97	[87]
							rK26	2	360	90–97	97–100	[13,90]
							rK28	2	675	89–100	91–99	[13,16]
							rK39sub	1	140	91	96	[91]
							rK9	2	217	78–96	74–90	[13,92]
							rKE16	1	412	99–100	100	[93]
							rKMP11	1	81	100	100	[73]
							rKO8	1	183	98	96	[94]
							rKRP42	3	746	94–97	98–100	[17,95,96]
							rLACK	1	146	97	84	[31]
							rLiHyS	1	103	100	100	[75]
							rPeroxidoxin	1	125	100	96	[97]
							rPHB	1	102	100	99	[98]
							rTR18	1	124	37	94	[99,100]
							SLA/ Lysate	7	1047	40–100	48–100	[31,39,74,75,98,101,102]
**other**	200-kDa fraction WB	1	60	97	100	[103]	A2 latex agglutination test	1	105	88	94	[104]
	60 kDa membrane-associated antigen WB	1	42	100	100	[105]	DAT	5	ca. 800	87–100	93–99	[10]
	DAT	20	ca. 4000	92–96	94–99	[10]	dot blot with SLA	1	126	100	100	[106]
	Dot RIA ConA specific antigen	1	28	100	100	[54]	IFAT	5	1910	80–92	91–100	[70,101,102,112,113]
	ESAs agar diffusion	1	70	69	100	[111]	rKE16 flow through test	1	599	99	not indicated	[28]
	IFAT	2	154	98–100	70–100	[61,114] [61]	SLA 16 kDa antigen WB	1	157	95	98	[115]
	IFAT amastigotes	1	70	100	91	[114]	SLA 70 kDa band WB	1	102	94	90	[117]
	WB SLA 12, 14 and 16 kDa band	1	95	64–100	100	[116]	SLA banding pattern WB	1	116	100 HIV -, 71 HIV +	100 HIV -, 73 HIV+	[118]
	WB rK26	1	27	100	100	[79]	polypeptides 40, 33, 17 WB	1	119	79–90	92	[120]
	WB rLepp12	1	55	100	100	[119]						
	**phase III**											
**Test format**	**antigen name**	**number of studies**	**number of specimens tested**	**reported sensitivity [%]**	**reported specificity [%]**	**ref**.						
**ICT**	rK26	1	352	21	100	[25]						
	rK28	1	285	95	98	[15]						
	rK39	20	3622	85–97	86–97	[6]						
	rKE16	1	219	77	96	[29]						
**ELISA**	B-ME ELISA	2	490	93–98	92–100	[32,33]						
	FML	1	402	100	96	[36,37]						
	rK28	1	168	99	96	[17]						
**other**	DAT	4	774	88–97	76–97	[10]						
	FAST	2	148	91–96	71–86	[6]						
	IFAT	5	1034	28–100	83–99	[89,107–110]						

**Table 2:** Characteristics of kinesin related antigens.

name	specification	molecular weight [kDa]	*Leishmania* species	*Leishmania* strain	geographical origin of *Leishmania* strain	reference
rK39	recombinant kinesin 39	39	*L*. *infantum*	MHOM/BR/82/BA-2,C1	Brazil	[8]
rDD8	rK39 homologue from *L*. *donovani* DD8 strain	29	*L*. *donovani*	MHOM/IN/DD8/1968	India	[81]
rK39sub	rk39 homologue from Iranian *L*. *infantum*	39	*L*. *infantum*	LON49	Iran	[91]
rKE16	rK39 homologue from Indian *L*. *donovani*	26	*L*. *donovani*	MHOM/IN/KE16/1998	India	[93]
rKO8	kinesin related antigen	35	*L*. *donovani*	Lo8	Sudan	[94]
rKRP42	K39 homologue in *L*. *donovani*)	42	*L*. *donovani*	MHOM/IN/DD8/1968	India	[96]
K9-K26-K39	chimeric antigen epitopes of K9, K26, and K39	17	*L*. *infantum*	not indicated	not indicated	[59]
rK28	chimeric protein composed of parts of *L*. *donovani* rK39, rK9 and rK26	28	*L*. *donovani*	not indicated	not indicated	[13]

Forty four tests representing 42 antigens are evaluated on ≤100 specimens in phase I studies, 57 tests representing 53 antigens were evaluated on >100-specimens in phase II studies, 10 tests representing 9 antigens, were evaluated in phase III studies. Thus 86 out of the 96 tests (89%) were evaluated in phase I and/or II only. Seven tests (6 antigens) in phase III are evaluated on less than 600 specimens, while 3 antigens/tests are evaluated on 601 to 5000 specimens and no antigen is evaluated on >5000 specimens.

Seventeen of the tests in phase I, 11 in phase II and 1 test in phase III (rK28 immunochromatographic test [ICT] from CTK) consistently have sensitivities above 94% and specificities above 97% in all studies analysed (green in Fig 2 and grey in Table 1).

### The characteristics of the antigens

Fifty-three antigens (59%) were expressed in *E*. *coli* or on phages or were synthetic peptides and thus do not contain eukaryotic glycosylated or lipid moieties. Two of the native antigens were analysed for glycosylation and do not contain a carbohydrate moiety. P32 and the 62 to 63 kDa protein were shown to have no glycosylation by acid-Schiff staining and the latter also does not bind Concanavalin A [30,68].

The remaining 34 (38%) are divided in three categories. In 14, the nature of the antigen could not be deducted from its isolation procedure (parasite lysate run on sodium dodecyl sulfate polyacrylamide gel electrophoresis (SDS-PAGE) and Western blot, antigen eluted form SDS-PAGE, acid extraction, isolation using a Lipidex-1000 column [34,41,44,48,63,104,118,121]) and no additional analysis has been performed to conclude on its nature. Five antigens bear a carbohydrate while it is not certain whether they bear a lipid moiety, such as the concanavalin-A specific antigen isolated by Bhattacharya [54], gp 70–2 which reacts with the monoclonal D2 and was sensitive to periodate treatment [55], two competition ELISAs using D2 and other monoclonal antibodies [46,50] and FML was found to be composed of sugars by colorimetry [36]. Fifteen antigens contain both a carbohydrate and a lipid moiety as they are made from whole parasites, whole parasite lysates, the entire secretome, the parasite membrane or lipophosphoglycan (LPG) [31,32,35,60,62,111,114,122].

**Fig 3:**
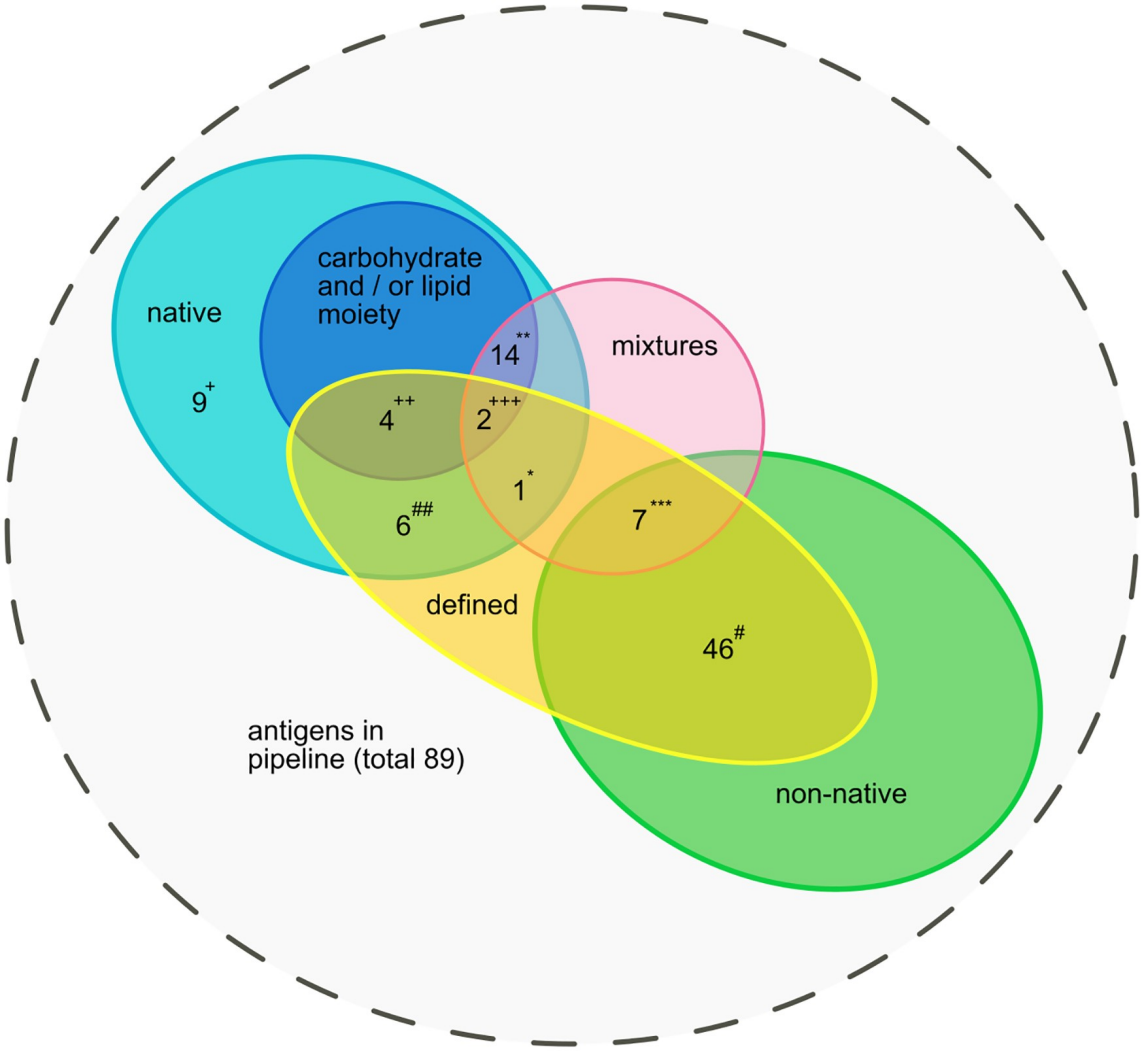
Single antigens and mixed antigen preparations grouped according to their origin (native versus non-native), the presence or absence of carbohydrate and/or lipid moieties, and their defined or undefined character. *crude *Leishmania* histone, **e.g. DAT, ***e.g. rK28, # e.g. rK39, e.g. BHUP 1–3, + e.g. P32, ++ e.g. LPG, +++ FML, monoclonal antibodies D2 + D13 + D14.

The second part of the analysis of the antigen characteristics is summarized in Fig 3. All 20 antigens with a known carbohydrate and or lipid moiety are native antigens. Fourteen are antigen mixtures of undefined identity, such as the DAT [122] and soluble leishmanial antigen (SLA) (e.g. [62]) which consist of a mixture of parasite molecules including carbohydrates and lipids. Two are antigen mixtures which can be considered as defined: FML [36,37] and the competition ELISA using the monoclonal antibodies D2, D13 and D14 to inhibit antibodies reacting with SLA [50]. Four antigens containing a carbohydrate and/or lipid moiety can be considered defined: lipophosphoglycan [31], concanavalin-A specific antigen [54], gp 70–2 [123] and the inhibition ELISA using the monoclonal antibody D2 (which is directed against gp 70–2) to inhibit antibodies reacting with SLA [46]. Among the antigen mixtures that lack a known carbohydrate or lipid moiety, 8 are defined. One is a native antigen (crude *Leishmania* histone (CLH) [48]) while the other 7 are non-native.

### Availability of data on evaluation with East African specimens

**Fig 4:**
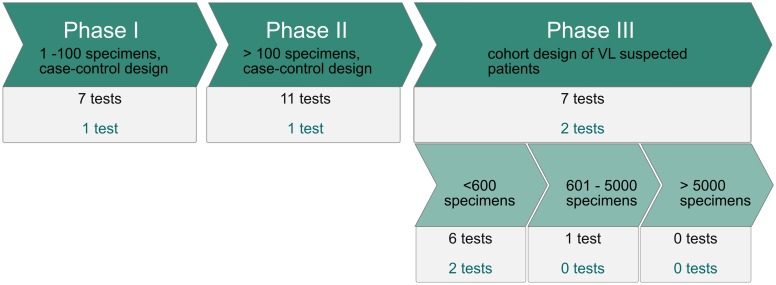
Position of serodiagnostic tests for VL along the different phases of the diagnostic development pipeline, based on their evaluation on East African specimens. Each test is defined by its antigen and the test format. The number of tests shown in black is the total number of tests that have been evaluated in each phase. The number of tests shown in black gives the total number of tests per phase, the number of tests shown in green gives the number of tests that meet the cut off of >94% sensitivity and >97% specificity for an ideal test defined by Boelaert *et al*, 2007 [18].

Nineteen tests, representing 19 (21%) antigens, were evaluated on East African specimens. Fig 4 and Table 3 show the position of these tests and antigens along the diagnostic development pipeline. Only Indirect Fluorescent Antibody Technique (IFAT) and the rK28 ICT were shown to be >94% sensitive and >97% specific when evaluated in phase III studies on respectively 104 and 285 East African persons [15,108]. The other tests in phase III are rK39 ICT, rKE16 ICT, beta-mercaptoethanol modified ELISA (ß-ME ELISA), DAT and FAST. They were evaluated on respectively 1692, 219, 490, 208 and 148 persons but showed lower sensitivities and/or specificities in at least one of the studies [6,10,29,32,33]. All the other tests and antigens have only been evaluated in phase I and II studies. Among those, the SLA in dot blot (evaluated on 126 specimens) and the 70 kDa soluble antigen in ELISA (evaluated on 42 specimens), were >94% sensitive and >97% specific [38,106].

**Table 3:** Detailed overview of serodiagnostic tests for VL in the diagnostic development pipeline tested on specimens from East Africa. For tests evaluated in multiple studies the lowest and highest values are taken for the sensitivity and specificity range. For the meta-analysis reports, the confidence intervals are considered as the range. Highlighted in grey are the tests that meet the cut off of >94% sensitivity and >97% specificity for an ideal test defined by Boelaert et al, 2007 [25].

	**phase I**						**phase II**					
**Test format**	**antigen name**	**number of studies**	**number of specimens tested**	**reported sensitivity [%]**	**reported specificity [%]**	**ref**.	**antigen name**	**number of studies**	**number of specimens tested**	**reported sensitivity [%]**	**reported specificity [%]**	**ref**.
**ICT**							rK28	3	629	94–99	81–100	[13,14]
							rKE16	1	750	37	98	[12]
**ELISA**	70 kDa soluble antigen	1	42	100	100	[38]	B-ME ELISA	1	314	93	96	[45]
	70-P2	1	33	57	100	[40]	Intact promastigote ELISA	1	135	100	not calculated	[58]
	Intact promastigote ELISA	2	161	95–100	88–97	[61,62]	r(g)p63	1	130	84	98	[72]
	peptides gp63	1	100	all 5: 97, P2+P4/P3: 100	all 5: 76, P2+P4/P3: 94	[71]	rK26	1	137	90	97	[13]
	rA2	1	55	70	100	[76]	rK28	1	137	97	96	[13]
	SLA/ Lysate	1	77	98	97	[62]	rK9	1	137	90	83	[13]
							rKO8	1	183	98	96	[94]
**Other**	IFAT	1	84	100	70	[3]	dot blot with SLA	1	126	100	100	[106]
							DAT	9	208	89–96	89–99	[10]
	**phase III**											
**Test format**	**antigen name**	**number of studies**	**number of specimens tested**	**reported sensitivity [%]**	**reported specificity [%]**	**ref**.						
**ICT**	rK28	1	285	95	98	[10]						
	rK39	9	1692	75–93	80–97	[6]						
	rKE16	1	219	77	96	[29]						
**ELISA**	B-ME ELISA	2	490	93–98	92–100	[32,33]						
**Other**	DAT	2	208	89–96	89–99	[10]						
	FAST	1	148	91	71	[6]						
	IFAT	1	104	100	99	[108]						

## Discussion

### Diagnostic development pipeline analysis

There is a multitude of publications on new diagnostic tests for VL, of which many report very high diagnostic accuracies (sensitivities and specificities up to 100%). While this seems very promising, diagnostic accuracies of tests are difficult to be compared to one another as they are not necessarily assessed on a comparable number and type of specimens. We analysed the level of evaluation and the diagnostic accuracies reported for every test published until 2018 to situate them in the diagnostic development pipeline and to facilitate comparisons between tests and antigens used in these tests. According to Boelaert *et al*. 2007 [18] the diagnostic development pipeline comprises three major phases defined by purpose and study design. Phase I and phase II follow a case-control design and deliver the proof-of-principle and intrinsic sensitivity and specificity of a diagnostic test, which are mostly defined by the antigen and the assay format. To validate the diagnostic accuracy of a test, it should undergo an evaluation in a prospective phase III study conducted on a larger number of representative samples of consecutively enrolled or randomly selected patients within the population [18,19]. Our analysis shows that 89% of the tests were evaluated in phase I and/or phase II. To a certain extent, this can be explained by the fact that underperforming candidates are not taken further along the development process. This is however not the case for 13 of the antigens in phase I and 10 of the antigens in phase II with observed sensitivities and specificities higher than 94 and 97% respectively that were not evaluated in the following phase. Why these antigens were not taken to the next level of evaluation cannot be explained by our data. It does however fit with the often described “valley of death” between discovery and commercialisation [124], a term that is used to describe the deficiencies in translating discoveries from (mostly academic) research into commercial products by the industry. There seems to be a lack of connectivity between the different stages of development, which hampers the advance of new diagnostics tools, leaving promising compounds on the back shelf. Moreover, it creates the impression of a vast variety diagnostic candidates, while they were never sufficiently evaluated to make conclusions on their performance. The number of extensively evaluated tests with high diagnostic sensitivity and specificity is thus very low, which can be explained by the correlation between the number of specimens and studies in which a test has been evaluated and its probability to be screened out for its insufficient diagnostic accuracy.

### Characteristics of antigen candidates

In a recent literature review on the nature of the antigen in the DAT, which is highly sensitive and specific for VL diagnosis across its geographical distribution, we concluded that alternative candidate antigens for VL serodiagnosis should be composed of a mixture of antigens that also contains a non-protein moiety and that can be produced recombinantly or synthetically [125]. From the present analysis, it appears that no antigen candidate with these characteristics exists in the diagnostic development pipeline for VL diagnosis.

Fifty three antigens are recombinantly expressed in *E*. *coli* or on phages or are synthetic peptides. These proteins and peptides do not undergo eukaryotic posttranslational modifications like glycosylation. Thus, epitopes based on carbohydrate and lipid moieties present on the original parasite components are not represented on these antigens. Of the remaining 36 antigens, only 20 certainly bear a carbohydrate and/or lipid moiety.

Among the 96 diagnostic tests analysed, we were able to identify 24 antigens in the diagnostic development pipeline that contain antigen or epitope mixtures.

Ideally, an alternative serodiagnostic test for VL should be manufactured at low cost and with a high level of standardisation. Therefore, using native antigens is less desirable since their production is usually expensive and inherently prone to considerable batch to batch variation. Even more so when the antigens are not defined in the mix of molecules used. We therefore analysed how many of the carbohydrate and / or lipid containing antigens and how many of the antigen mixtures are native and whether they are composed of more or less defined molecules, as opposed to being crude parasite extracts or whole cell antigens. We found that all 20 antigens that contain a non-protein moiety are native and 14 are undefined. Sixteen antigens with carbohydrate and/or lipid moieties are antigen mixtures, 14 of which are undefined. Only 7 out of the 24 antigen mixtures are not native, i.e. consist of recombinants or peptides.

### Candidates for East Africa

Considering that a reliable point of care (POC) test for VL in East Africa is not secured yet, we analysed the tests in the diagnostic development pipeline that have been tested on sera from East African VL patients. We found that only the IFAT and the rK28 ICT [15,108] have shown a high diagnostic accuracy (sensitivity and specificity above 94 and 97% respectively) in cohorts of VL suspected patients in East Africa. All other candidates with high diagnostic accuracies on East African specimens (dot blot with SLA [106] and a ELISA with a 70 kDa soluble antigen [38]) were evaluated in case-control designed studies and might be screened out upon further evaluation.

There is 1 non-native, defined antigen with high diagnostic accuracy on East African specimens: the rK28, which incorporated in an ICT has been evaluated on 285 persons in phase III. Apart from this promising candidate the pipeline of antibody detection tests for VL detection in East Africa almost empty.

### Limitations of the study

Our analysis was limited by the fact that we only searched one database (PubMed). The data extraction was performed by one investigator only, which might have led to errors. We did not consider the risk of bias on individual study level which might have impacted the analysis on the diagnostic sensitivities and specificities of the tests. However our aim was to show the landscape of existing tests and tendencies within it, which we believe is not hindered by the limitations of the study, as the search and analysis were performed systematically.

### Conclusions and way forward

With our analysis on the development pipeline of new serodiagnostic POC tests for VL especially for East Africa, we identified five major problems:

(i) The number of tests and antigens that have been extensively evaluated in phase III studies is low, with more than three quarters of all tests having been evaluated in phase I and II. Especially in Eastern Africa where antigen diversity is high compared to South-Asia [126] we consider it essential to conduct a large-scale evaluation on a broad panel of sera from different geographic origin, before concluding on the diagnostic potential of a test. We recommend including a sufficient number of specimens from Eastern Africa in the downstream evaluation of any new POC test for VL showing high diagnostic accuracy in early phases of development. A major obstacle for this is the limited number of and access barriers to reference serum archives (ii) Not enough emphasis is put on non-protein moieties of candidate antigens, with only 20 of the 89 antigens bearing a defined carbohydrate or lipid moiety. (iii) The fact that all antigens with carbohydrate and/or lipid moieties and 17 of the 24 mixed components antigens are native, poses a problem for the eventual commercialisation in terms of production cost and batch-to-batch variation. For well identified antigens or antigen mixtures, eukaryotic expression systems such as developed in *Leishmania tarentolae*, *Pichia pastoris* or *Spodoptera frugiperda* should be envisioned, as they allow to produce recombinant proteins with eukaryotic post-translational modifications, including glycosylation [127–129]. For undefined antigens, screening of peptide libraries could be considered to select short peptides (mimotopes) that mimic native carbohydrate and lipid epitopes. Umair and co-workers proved this concept by using phage display technology to discover a mimotope that can replace the glycan epitope on the surface of parasitic nematode larvae [130]. (iv) Mixtures of antigens are not sufficiently taken into account; only 7 candidate recombinant antigens consist of a mixture of several compounds. The development of non-native antigen mixtures should be extended. We see two possibilities for creating antigen mixtures: a) chimeric recombinant antigens such as rk28 [13], Q protein [73], the chimeric antigen with epitopes of *L*. *infantum* K9, K26, and K39 [59] and the synthetic peptide P1P2 which consists of two epitopes of the hypothetical protein NCBI reference: XP_003861458,1 [26] b) combinations of several antigens such as the mixture of synthetic peptides derived from gp63 on human serum albumin [71], of synthetic peptides from antigens A2, NH, LACK and K39 [69] and the OrangeLife ICT which is based on an association of rK39 and rK28 [22]. (v) 70% of all carbohydrate and lipid containing antigens and 71% of all antigen mixtures are non-defined. Aiming at the development of alternative diagnostic test for VL in East Africa that complies with the ASSURED criteria, investigations should be undertaken to identify these reactive epitopes and to replace them by recombinant or synthetic antigens.

## Supporting information

S1 Table Key information from the studies included in this review. Ag: antigen, AIDS: acquired immune deficiency syndrome, ALA: amoebic liver abscess, ß -ME: beta-mercaptoethanol, CI: confidence interval, CIC: circulating immune complex, CL: cutaneous leishmaniasis, CP: cysteine proteinase, *d*: *donovani*, DAT: Direct Agglutination Test, *E*: *Escherichia*, EA: East Africa, ELISA: enzyme-linked immunosorbent assay, FACS: fluorescence-activated cell sorting, FCS: foetal calf serum, HAT: human African trypanosomiasis, HB: hepatitis B, HCV: hepatitis C virus. HEC: healthy endemic control, HIV: human immunodeficiency virus, HSA: human serum albumin, HNEC: Healthy non-endemic control, ICT: immunochromatographic test, IFAT: indirect fluorescent antibody technique, ISC: Indian subcontinent, kDa: kilodalton, *L*: *Leishmania*, mAb: monoclonal antibody, MCL: mucocutaneous leishmaniasis, NA: not applicable, OD: other disease, PEG: polyethylenglycol, r: recombinant, SDS-PAGE: sodium dodecyl sulfate polyacrylamide gel electrophoresis, SLA: soluble leishmanial antigen, TB: tuberculosis, VL: visceral leishmaniasis, WB: Western blot.
(XLSX)

S1 Checklist PRISMA 2009 Checklist. (DOC)
